# Uremic Cardiomyopathy: A New Piece in the Chronic Kidney Disease-Mineral and Bone Disorder Puzzle

**DOI:** 10.3389/fmed.2018.00206

**Published:** 2018-07-24

**Authors:** Paulo G. de Albuquerque Suassuna, Helady Sanders-Pinheiro, Rogério B. de Paula

**Affiliations:** ^1^Laboratory of Experimental Nephrology and Interdisciplinary Nucleus of Laboratory Animal Studies, Federal University of Juiz de Fora, Juiz de Fora, Brazil; ^2^Interdisciplinary Center for Studies, Research and Treatment in Nephrology, Federal University of Juiz de Fora, Juiz de Fora, Brazil

**Keywords:** chronic kidney disease, chronic kidney disease-mineral and bone disorder, uremic cardiomyopathy, Klotho, FGF23

## Abstract

Cardiovascular diseases are the main cause of death in chronic kidney disease (CKD) patients. In dialysis patients, sudden cardiac death accounts for 40% of all deaths. In these patients, sudden cardiac death is usually secondary to an underlying cardiomyopathy, which is clinically identified by the high prevalence of left ventricular hypertrophy and the resultant mechanical and electrical dysfunction. CKD-related cardiomyopathy has a multifactorial pathophysiology. Recent evidence has highlighted the central pathophysiological role of chronic kidney disease-mineral and bone disorder (CKD-MBD) with hyperphosphatemia and high fibroblast growth factor 23 (FGF23) levels in these patients. Further, since CKD is known to be an αKlotho deficiency state, experimental studies have demonstrated that the deleterious effects of FGF23 can be minimized by reestablishing adequate soluble Klotho levels. Herein, we present a review that addresses not only the development of the understanding of CKD-related cardiomyopathy pathophysiology, but also explores the recent data that identify the triad of hyperphosphatemia, high FGF23 levels and αKlotho deficiency as playing a central role on it. Taken together, the data suggest that the uremic cardiomyopathy can be considered a new piece in the CKD-DMO puzzle.

## Introduction

Cardiovascular disease is the leading cause of morbidity and mortality in chronic kidney disease (CKD) (1). Left ventricular hypertrophy (LVH) is the most prevalent cardiac abnormality in these patients and is closely related to mortality (2, 3). This adverse cardiac outcome is consequence of the underlying CKD-related cardiomyopathy, which has been termed “Uremic Cardiomyopathy” (4–6). However, the pathogenesis of uremic cardiomyopathy is multifactorial and is poorly understood (7).

Despite advances in dialysis treatment and in the improved management of hypertension, hypervolemia, anemia, and chronic kidney disease—mineral and bone disorder (CKD-MBD), patients with CKD continue to have abnormal myocardial remodeling, which results in the persistently high rates of cardiovascular events and mortality (8).

In the last two decades, two new components of CKD-MBD have been identified: the hormone FGF23, and its cofactor, αKlotho. FGF23 is deleterious to the myocardium, while αKlotho is protective (9–17). Although αKlotho is an obligatory cofactor for FGF23 action as the primary phosphaturic hormone in phosphorus homeostasis, the two seem to have independent and antagonistic effects on the myocardium (18).

The impact of CKD-MBD has already expanded beyond the skeleton itself, once its direct involvement in vascular calcification had been demonstrated (19). Currently, the identification of the triad of hyperphosphatemia, αKlotho deficiency, and elevated FGF23 levels as being central in the pathophysiology of uremic cardiomyopathy, unravels uremic cardiomyopathy as a new piece in the CKD-MBD puzzle, paving the way for more specific therapeutic possibilities for these patients.

## Cardiovascular disease in chronic kidney disease: the role of left ventricular hypertrophy

Only a third of CKD patients survive until they receive dialysis owing to the progressive increase in cardiovascular risk, secondary to glomerular filtration rate deterioration (20).

The overall outcomes worsen following the initiation of dialysis. Foley et al. (21) in their classic study published in 1998, demonstrated that the risk of death for patients on dialysis was 10–100 times higher than that expected for age-matched general population. Furthermore, this trend has remained unchanged even after a decade of advances in dialysis treatment (8).

The reason for the very high cardiovascular risk in CKD patients has already been widely addressed by several authors, and appears to be related to the synergic interaction between traditional cardiovascular risk factors (age, hypertension, dyslipidemia, obesity, diabetes, tabaco use, male gender, physical inactivity, positive family history) and non-traditional cardiovascular risk factors (homocysteine, lipoprotein (a), small dense LDL, fibrinogen, C-reactive protein, hypercoagulability, ACE genotype, depression) altered by CKD and new CKD-related risk factors (uremic toxins, anemia, hypervolemia, oxidative stress, inflammation, insulin resistance, CKD-MBD) ensuing a faster progression of CVD and increasing the number of cardiovascular events and mortality (22, 23).

Sudden cardiac death (SCD), which accounts for 40% of all causes of death among dialysis patients, is the main cardiovascular cause of death in CKD, and outweighs heart failure, acute myocardial infarction, and stroke (24).

**Figure 1:**
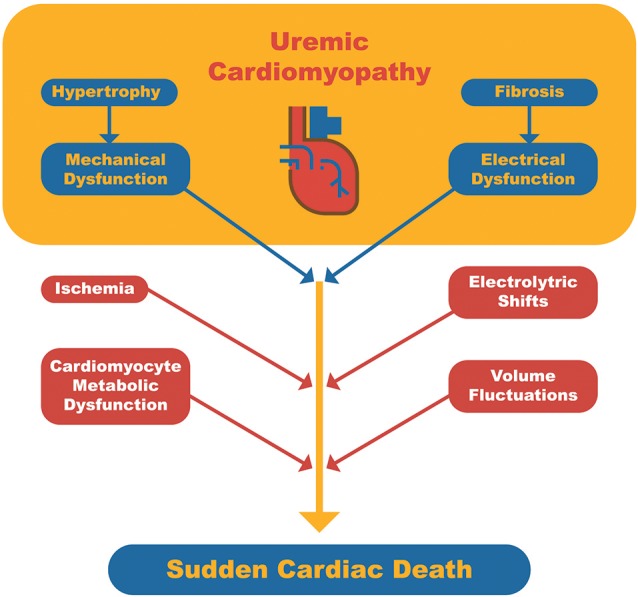
Schematic representation of sudden cardiac death pathophysiology in dialysis patients. Uremic cardiomyopathy serves as the arrhythmogenic substrate and triggers related to dialysis or cardiomyocyte metabolism may lead to a fatal arrhythmia.

Although SCD may occur in individuals with structurally normal myocardium, it is commonly observed in patients with underlying cardiomyopathy, which serves as a substrate for triggering fatal arrhythmia (25). In the general population, the most frequent trigger for SCD is acute myocardial ischemia. In contrast, in dialysis patients, a number of other triggers or risk factors may be identified: anemia, CKD-MBD (high parathyroid hormone [PTH] levels, hypo or hypercalcemia and hyperphosphatemia), fast electrolyte shift, chronic volume overload, inflammation, coronary artery disease, autonomic dysfunction (sympathetic hyperactivity and baroreflex dysfunction), atrial fibrillation, heart failure with systolic dysfunction, and left ventricular hypertrophy (LVH) (Figure 1) (26).

When evaluating in CKD patients the existence of a cardiomyopathy that serves as an arrhythmogenic substrate, the high prevalence of LVH draws attention first. LVH is present even in very early CKD stages, reaching a prevalence of up to 65% in pre-dialysis patients (3). In addition, the prevalence of LVH, likewise the cardiovascular risk, parallels the progression of CKD. LVH is seen in about 75% of adult patients and 69% of pediatric patients at the onset of dialysis, and almost 100% of patients after 5 years on dialysis (27–29).

LVH has a high negative impact on the survival of dialysis patients. Serial echocardiographic evaluation of dialysis patients has demonstrated a strong positive correlation between the presence and severity of LVH, and cardiovascular morbidity and mortality (3, 28). In their work published in 2004, Zoccali et al. (29) demonstrated that the progression of LVH in dialysis patients was associated with mortality and cardiovascular events, regardless of the baseline ventricular mass or other cardiovascular risk factors. Thus, patients with a greater increase in ventricular mass had poorer prognosis. This observation reflects the tissue level derangements in the myocardium of CKD patients, with LVH being the clinical expression of the underlying pathophysiological process that contributes to the development of a CKD-related cardiomyopathy, best known as uremic cardiomyopathy (6).

## CKD-related cardiomyopathy (uremic cardiomyopathy)

In 1967, Bailey et al. were the first to describe a cardiomyopathy related to uremia. They highlighted the presence of significant cardiomegaly and the role of protein restricted diet in reversing it, based on data from four uremic patients with very high creatinine and urea levels maintained on conservative treatment (30).

Almost a decade later, in 1975, Ianhez et al. from the University of São Paulo, reported seven CKD patients with a clinical picture of cardiomyopathy and pericarditis that improved either with hemodialysis or renal transplantation (31).

The use of terms such as “uremic heart” or “uremic cardiomyopathy” emerged following these reports and triggered considerable debate about its existence and its possible pathophysiological mechanisms (32). The initial candidate factors suspected of contributing to uremic cardiomyopathy were uremic toxins, chronic hypervolemia, anemia, circulating catecholamine excess, L-carnitine deficiency, and secondary hyperparathyroidism (6).

Based on these historical studies, we concluded that the term uremic cardiomyopathy was coined about five decades ago, coinciding with the introduction of dialysis treatment. The usual clinical picture was of severe uremia accompanied by cardiomegaly, systolic dysfunction and pericarditis (4, 33).

Advances in dialysis treatment were accompanied by a significant cardiologic improvement and increase in patient survival. Dialysis contributed to attenuation of uremia as well as correction of hypervolemia, hydro-electrolytic balance and acid-base disorders, nutritional improvement and partial correction of anemia (32). Although it is clear that the pathogenesis of CKD-related cardiomyopathy is multifactorial, it was observed that despite the correction of several contributory factors, LVH continued to be highly prevalent and precocious in patients with CKD (6).

## The role of CKD-MBD in CKD-related cardiomyopathy

**Figure 2:**
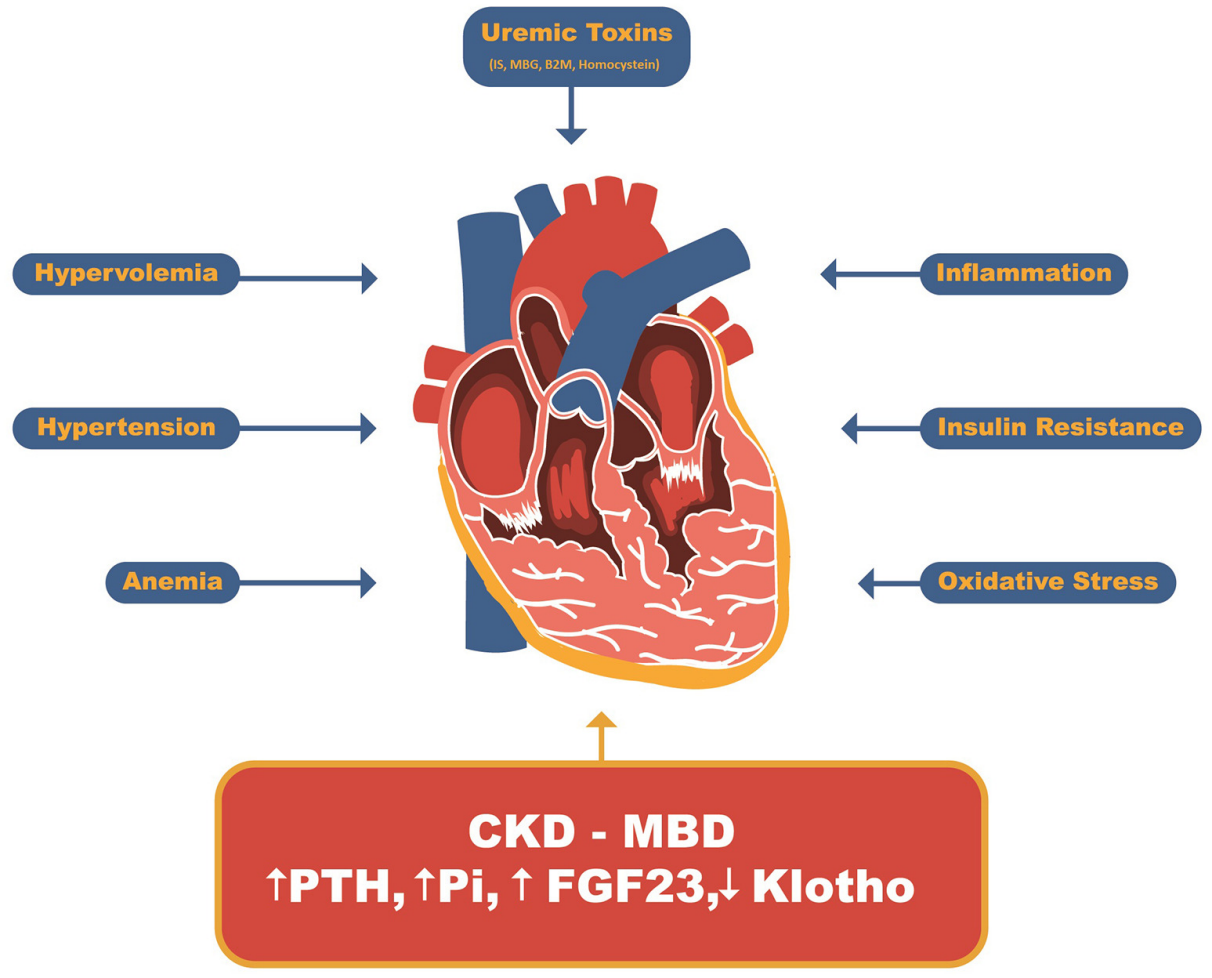
Uremic Cardiomyopathy related factors. Beyond uremic toxins such as indoxil sulfate, marionobufagenin, β2-microglobulin and homocysteine and other CKD-related factors such as hypervolemia, hypertension, anemia, insulin resistance, inflammation and oxidative stress, the CDK-MBD has recently been stood out as major player related to uremic cardiomyopathy. IS, Indoxyl sulfate; MBG, Marionobufagenin; P, Phosphate; B2M, β2-Microglobulin; PTH, parathyroid hormone; FGF23, Fibroblast growth factor 23.

The role of CKD-MBD in the pathophysiology of uremic cardiomyopathy, owing to its strong correlation with cardiovascular events and mortality, was recognized following the advances in dialysis treatment and correction of major uremia-related abnormalities (Figure 2) (12, 34, 35).

Initially, secondary hyperparathyroidism was found to be associated with vascular calcification as well as myocardial hypertrophy and cardiac dysfunction (6). This association is known to occur even in patients with primary hyperparathyroidism, wherein high PTH levels correlate with ventricular mass and the degree of diastolic dysfunction, both of which are reversed following treatment for hyperparathyroidism (36). Further studies have also demonstrated a higher prevalence of systemic arterial hypertension in patients with primary hyperparathyroidism. Experimental studies have shown an increase in calcium influx into cardiomyocytes, and elevated systemic aldosterone levels, with consequent activation of cardiomyocyte mineralocorticoid receptors induced by high PTH levels (37–39).

Recent evidence has provided us with a better understanding regarding the role of hyperphosphatemia in vascular calcification and has identified the central role of phosphate in the CKD-MBD pathophysiology. Phosphotoxicity is now considered to be the main cause of cardiovascular mortality (34). Vascular calcification is currently understood to be an active process regulated by cells, wherein ectopic deposition of calcium and phosphate salts occurs, especially in the muscular layer of arteries and heart valves (19). Hyperphosphatemia drives vascular calcification by acting directly on the type III sodium-dependent phosphate cotransporter (PiT-1) of vascular smooth muscle cells (VSMC), elevating intracellular phosphate levels, and activating bone formation-related gene expression (19).

In addition, hyperphosphatemia is also toxic to endothelial cells and results in endothelial dysfunction, release of endothelial membrane microparticles with strong procoagulant action, and induction of endothelial cell apoptosis (40). This effect on endothelial cells is also PiT-1 dependent (41).

However, there is no definitive evidence of a hyperphosphatemia-induced direct effect on cardiomyocytes. Although, several studies have demonstrated cardiomyocyte hypertrophy and myocardial fibrosis in animals fed with a high-phosphate diet, the levels of circulating FGF23 and αKlotho were not measured (40). In cell cultures, high phosphate levels appear to induce cardiomyocyte apoptosis, increased VSMC proliferation, and markedly enhanced migration. These may suggest hyperphosphatemia-mediated induction of myofibroblast transformation (42, 43).

In summary, hyperphosphatemia in CKD directly induces coronary and systemic vascular calcification, calcification of cardiac valves, and endothelial cell dysfunction and apoptosis. It may indirectly lead to LVH by inducing hypertension, increased pulse pressure, and cardiac afterload. Evidence suggests that it may induce cardiomyocyte apoptosis, which may contribute to remnant cardiomyocyte hypertrophy and increase myocardial fibrosis by inducing VSMC migration from vessels and their transformation into myofibroblasts. This may explain the areas of perivascular fibrosis commonly seen in CKD-related cardiomyopathy (40, 42, 43).

## The role of FGF23 in CKD-related cardiomyopathy

FGF23 belongs to the endocrine FGFs subfamily and is expressed in humans by the FGF23 gene located on chromosome 12. It is a 227 amino acids peptide that is secreted mainly by osteocytes (44). FGF23 mutations were first described in 2000 in relation to the rare autosomal dominant hypophosphatemic rickets syndrome (ADHR) (45). ADHR is caused by uncontrolled FGF23 activity resulting in excessive renal phosphate loss, hypophosphatemia, very low levels of 1,25-dihydroxyvitanin D, osteomalacia, and rickets. The other end of FGF23 activity spectrum is represented by the familial tumor calcinosis syndrome. In this syndrome, there is a loss of FGF-23 activity resulting in hyperphosphatemia, elevated 1,25-dihydroxyvitamin D levels, ectopic and vascular calcifications, and premature death. These two rare clinical syndromes provided the first supporting evidence of a physiological role of FGF-23 in phosphate homeostasis (44). Currently, more than ten FGF23 gain of function diseases were already described and are grouped as FGF23-related hypophosphatemic diseases (46).

As FGF23 shows low affinity to FGF receptors, it needs membrane-bound αKlotho as cofactor, which makes the type 1c FGF receptor (FGFR1c) highly specific for FGF23 (47). Following the receptor binding and activation of intracellular signaling, the externalization of type 2a/c sodium-dependent phosphorus cotransporters (NaPi-2a and NaPi-2c) on the proximal tubule's brush border membrane is blocked, resulting in decreased phosphate reabsorption from the glomerular filtrate and increased phosphaturia (44, 48).

In addition to its phosphaturic effect, FGF23 also blocks 1α-hydroxylase and enhances 24-hydroxylase activity. This results in a reduction in the synthesis of 1,25-dihydroxyvitamin D and an increase in its degradation, eventually leading to a reduction in intestinal phosphorus absorption. Finally, FGF23 reduces serum PTH levels and blocks its secretion, which reduces bone resorption and the release of phosphate into the circulation (48).

In CKD, the reduction in the number of nephrons and the tendency toward positive phosphate balance results in a compensatory increase in FGF23 levels to cause phosphaturia, and to decrease intestinal phosphate absorption, and bone phosphate release. Although, hyperphosphatemia is observed only in advanced CKD, serum FGF23 levels start rising early in the course of the disease, and reach extremely high levels in patients on dialysis (48).

Paradoxically, the expected beneficial effect of the compensatory increase in circulating levels of FGF23 to maintain phosphorus homeostasis contrasts with multiple observational data correlating elevated serum levels of FGF23 with cardiovascular and all-cause mortality in patients with CKD, especially those on dialysis. In addition, this higher mortality appears to be independent of serum phosphorus levels, suggesting a phosphate-independent mechanism (9–11).

The observation of a positive correlation between circulating FGF23 levels and mortality in CKD was followed by the identification of a positive correlation between FGF23 levels and the prevalence of LVH in dialysis patients. This led to the hypothesis that FGF23 may directly induce LVH, and therefore, increase mortality (11, 49).

In 2011, Faul et al. demonstrated that FGF23 causes hypertrophy of isolated cardiomyocytes by activating the calcineurin/NFAT signaling pathway. Although mediated by an FGF receptor, this action was independent of αKlotho cofactor. In addition, it was demonstrated *in vivo* that injection of FGF23 resulted in LVH. These findings revealed a causal role for FGF23 in the pathogenesis of LVH in CKD and suggest that chronic and extremely high FGF23 levels in dialysis patients may contribute to the development of LVH and mortality in this population (13).

In 2012, Shalhoub et al. analyzed the effect of long-term neutralization of FGF23 by anti-FGF-23 monoclonal antibody in an experimental CKD model. Surprisingly, although FGF23 neutralization improved serum PTH and 1,25-dihydroxyvitamin D levels, there was no impact on the parameters concerning myocardial hypertrophy. Furthermore, treated animals were found to have hyperphosphatemia, increased vascular calcification, and higher mortality. In this study, because of the high efficiency of the monoclonal antibody used, circulating FGF23 levels were drastically reduced to “sub-physiological” levels, what may explain the negative results of this therapeutic strategy (50).

Existing evidence suggest that although very high levels of FGF23 in a CKD scenario may be deleterious to the myocardium, implying in a maladaptation, at a “physiological” levels for a CKD scenario, FGF23 is fundamental for protecting the body from hyperphosphatemia-related cardiovascular injury (48).

Recently, human clinical trials involving FGF23-related hypophosphatemic diseases patients—X-linked hypophosphatemic rickets (XLHR) and Tumor-induced osteomalacia (TIO)—receiving a fully human recombinant IgG1 monoclonal antibody anti-FGF23 (burosumab) is ongoing (51). Results from phase 1 and 2 studies using burosumab to treat adults and children with XLHR showed biochemical, hormonal and skeletal improvement, but there are no data related to cardiac outcomes due the short follow-up (52, 53). There is also no data demonstrating an increased cardiovascular risk or a higher prevalence of LVH in patients with FGF23-related hypophosphatemic diseases, but this is not expected since there is no hyperphosphatemia and CKD. Thus, in these patients, serum FGF-23 levels are generally much lower than in dialysis patients, and both renal and soluble αKlotho levels are expected to be close to normal (54). Likewise, a higher risk of cardiovascular mortality is not expected in patients with XLHR or TIO in treatment with burosumab, since hyperphosphatemia is avoided.

**Figure 3:**
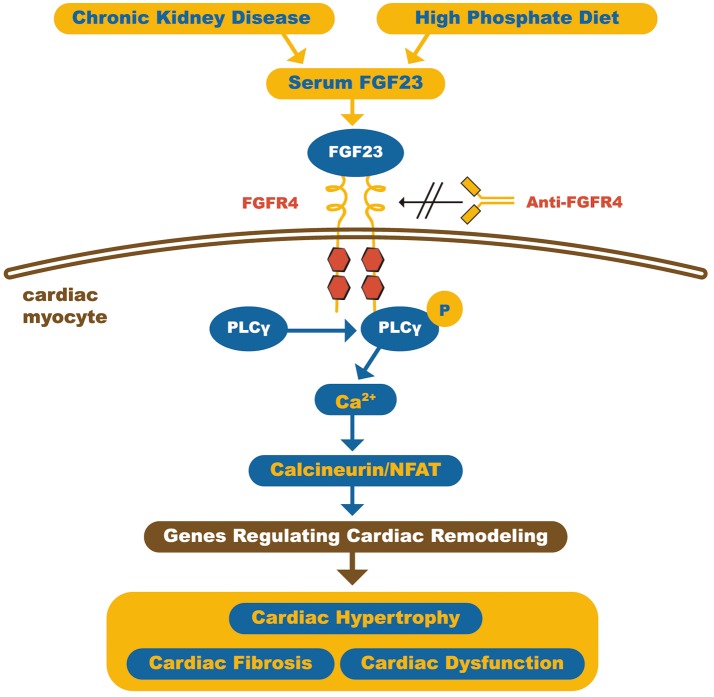
Schematic representation of how FGF23 can induce cardiomyocyte hypertrophy. The high circulating FGF23 levels in CKD patients can activate FGFR4 in cardiac myocytes and, through calcineurin/NFAT signaling pathway, activates cardiac remodeling genes, leading to hypertrophy and fibrosis. On the other hand, FGFR4 blockade by anti-FGFR4 can attenuate cardiac hypertrophy. FGF23, Fibroblast growth factor 23; FGFR4, type 4 Fibroblast growth factor 23 receptor; PLCγ, Phospholipase Cγ; NFAT, Nuclear factor of activated T-cells. Adapted from Grabner et al. (55).

Grabner et al. recently demonstrated that FGF23 acts on the myocardium through type 4 FGF receptor (FGFR4) activation in an αKlotho independent fashion. This involves activation of the PLCγ/Calcineurin/NFAT pathway and induces hypertrophy. Furthermore, it was also shown that blocking the FGFR4 receptor with monoclonal antibodies protected against FGF23-induced LVH, without loss of physiological effects of FGF23 (Figure 3) (55).

In summary, FGF23 plays an important role in CKD-related cardiomyopathy, and myocardial FGFR4 blockade appears to be a promising therapeutic target for it prevention.

## The role of αKlotho in CKD-related cardiomyopathy

The Klotho gene was accidentally identified by Kuro-o in 1997, when during an attempt to generate a spontaneously hypertensive transgenic mice lineage, a completely unexpected premature aging phenotype animal lineage resulted (56).

These genetically modified animals developed multiple phenotypic alterations that resembled premature aging including growth retardation, hypogonadotropic hypogonadism, rapid thymus atrophy, skin atrophy, sarcopenia, vascular calcifications, osteopenia, pulmonary emphysema, cognitive deficit, hearing loss, motor neuron degeneration, and premature death within 2 months of age (56).

In contrast, the transgenic mouse that overexpressed this gene, survived for about 30% longer than the wild type. Therefore, this gene was initially recognized as an aging process-related gene that increased the life span when overexpressed and decreased it when blocked. Consequently, it was given the name of the goddess Klotho, one of the three goddesses of Greek mythology responsible for spinning the thread of life (56).

The Klotho gene encodes the αKlotho protein, and is located on chromosomes 5, 12, and 13 in mice, rats, and humans, respectively, and has high homology between species (57). Three forms of αKlotho are found: transmembrane full-length αKlotho, soluble αKlotho and secreted αKlotho (57).

Transmembrane αKlotho is a type 1 single-pass transmembrane protein with 1014 (≈ 130 kDa) amino acids (aa). The N-terminal portion (1–33 aa) is hydrophobic and induces its translocation from the cytosol to the membrane. The transmembrane portion exhibits 20 aa, with a small intracellular portion of 9 aa with no known activity. The extracellular portion is composed of two subunits KL1 and KL2 (≈ 550 aa) with high homology between them (58, 59).

The transmembrane αKlotho can be cleaved by membrane-anchored proteases (ADAM10, ADAM17) and released as soluble αKlotho. The secreted αKlotho protein is the result of alternative splicing of the αKlotho gene mRNA and is composed of the KL1 subunit with ≈ 680 aa (58).

The αKlotho protein is expressed in a few tissues and organs, including the kidneys, parathyroid glands, choroid plexus, pancreatic islets, gonads, pituitary gland and sinoatrial node (60). The kidney is the organ with the highest expression of αKlotho and is the systemic source of soluble αKlotho in blood and urine. In addition, the kidney is also the main clearance site of circulating αklotho (60, 61).

In the kidney, both proximal and distal convoluted tubules express αKlotho in abundance on the basolateral side. However, to function as FGF23 co-receptor, it requires to form a binary complex with the FGF1c receptor that confers a high affinity for FGF23 (61). In addition to its function as FGF23 co-receptor, soluble αKlotho has many FGF23-independent pleiotropic actions, and acts in an autocrine, paracrine, and endocrine fashion (62).

In the kidneys, the soluble and membrane αKlotho expressed in tubular cells are eliminated in the urine through transepithelial transcytosis. Once in the tubular lumen, soluble αKlotho modulates the function of TRPV5 in favors of calcium reabsorption, ROMK1 that increases potassium efflux, and type 2a/c and type 3 NaPi co-transporters that are already expressed in the membrane, decreasing phosphate absorption (62).

**Figure 4:**
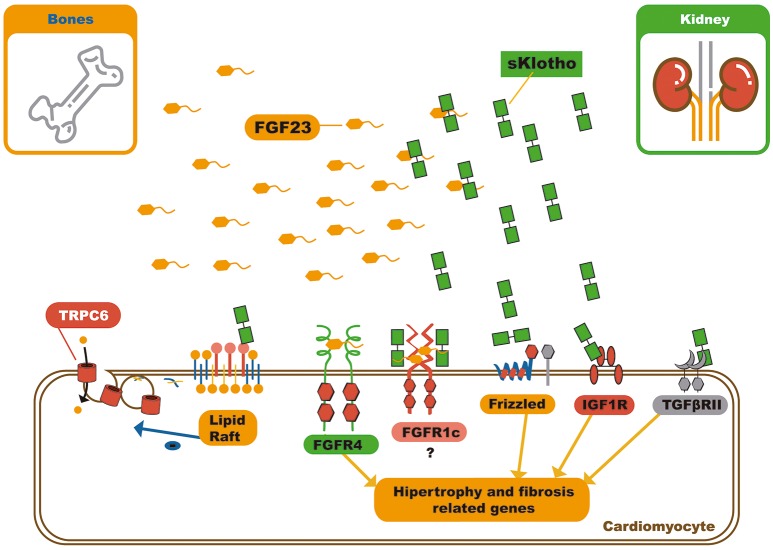
Schematic representation of soluble klotho pleiotropic functions and FGF23 – sKlotho interactions. Soluble klotho can modulate several signaling pathways by receptor or soluble factor biding. In relation to circulating FGF23, soluble klotho can bind it and act in three different ways: First, as decoy receptor leading to FGF23 inactivation or excretion; second, as a bona fide co-receptor, favoring FGF23 biding to FGFR1c, and third, soluble klotho can also serve as a on demand co-receptor to FGFR1c on cardiomyocyte membrane, changing the signaling pathway through FGF23 can act on myocardium. FGF23, Fibroblast growth factor 23; FGFR4, Type 4 fibroblast growth factor receptor; NFAT, Nuclear factor of activated T-cells; FGFR1c, Type 1c fibroblast growth factor receptor; IGF1R, Insulin growth factor 1 receptor; TGFβRII, Type II transforming growth factor β.

The pleiotropic effects of soluble αKlotho include, its interference in many signaling pathways like the insulin/IGF-1 (63), TGF-β (64), WNT (65, 66), and angiotensin II signaling pathways, in addition to protection against oxidative stress (Figure 4) (67, 68).

So far, no specific receptor for soluble αKlotho has been identified. Therefore, the mechanism by which soluble αKlotho acts as a hormone is poorly understood. The impossibility of soluble αKlotho to exhibit enzymatic activity, which was expected due to its high homology with β-glycosidases, has been clearly demonstrated. Consequently, its “endocrine” effects are believed to be due to its ability to bind to various proteins and receptors, and modulating their function without enzymatic action (69).

Dalton et al. demonstrated that soluble αKlotho binds to the monosialogangliosides present in membrane lipid rafts microdomains and alters the local lipid organization. This further lowers the capacity for endocytosis, and down-regulates raft-dependent PI3K/Akt signaling. This mechanism of action grants soluble αKlotho the ability to reduce externalization of the transient receptor potential cation channel subfamily C member 6 (TRPC6) on the cardiomyocyte membrane. It can also modulate several other signaling pathways (Figure 4) (70).

Although the myocardium does not express αKlotho, it has a high expression of FGF receptors, which on stimulation induce myocardial hypertrophy and fibrosis. As previously mentioned, FGF23 can act independently of αKlotho by activating FGFR4 receptors to induce myocardial hypertrophy by activation of the NFAT/calcineurin pathway. However, this action does not appear to occur in the presence of normal levels of soluble αKlotho (13, 17).

In 2012, Xie et al. published the first work that demonstrated the protective effect of αKlotho on myocardial hypertrophy and fibrosis in an isoproterenol-induced LVH model. They demonstrated the ability of αKlotho to reduce TRPC6 externalization in the cardiomyocyte membrane. TRPC6 refers to calcium channels that are activated by myocardial wall stress. These channels function as mechanosensors and induce hypertrophy by activating the calcineurin pathway (14). Subsequently, in 2013, Song et al. used the same model of isoproterenol-induced LVH and demonstrated that αKlotho replacement reduces isoproterenol-induced cardiomyocyte apoptosis by blocking the p38/MAPK signaling pathways and JNK kinases (71).

Following these two studies that demonstrated the protective action of αKlotho on the myocardium in LVH non-CKD models, two studies were published in 2014 that assessed the protective action of αKlotho on the myocardium in experimental CKD model. In these studies, LVH correlated with αKlotho deficiency state, hyperphosphatemia, FGF23 levels and age. Moreover, even after correcting hyperphosphatemia due to dietary phosphate restriction, and consequently reducing FGF-23 levels, LVH persisted in αKlotho deficiency CKD animals. This finding leads to the conclusion that there is a protective αKlotho effect, independent of phosphate and FGF23 levels. The protective effect of αKlotho was driven by its ability to block the hypertrophy and fibrosis induced by TGF-β1 and angiotensin II (15, 17).

In a more recent study, Yang et al. observed an inverse correlation between circulating αKlotho and indoxyl sulfate (IS) levels, and a positive correlation between IS levels and LVH, in both CKD patients and experimental CKD models. Additionally, recombinant αKlotho replacement was found to prevent LVH in CKD mice and block cardiomyocyte hypertrophy *in vitro*. This action was achieved by blocking oxidative stress and inhibiting the ERK 1/2 signaling pathway (16).

Finally, Yu et al. demonstrated that, *in vitro*, αKlotho was able to inhibit angiotensin II-induced hypertrophy in neonatal rat cardiomyocytes by down-regulating AT1 receptors and suppressing the AT1R/β-catenin signaling pathway (72).

Since the kidney is the main source of systemic αKlotho, any reduction in nephron number and decrease in GFR is followed by a progressive decline in circulating αKlotho levels (61). Thus, end-stage renal disease is an α-klotho deficiency state characterized by clinical and laboratory abnormalities that include high levels of FGF23, presence of hyperphosphatemia, vascular calcification, LVH, bone disease, premature aging, and high mortality (61). This raises the possibility that αKlotho deficiency may be the primary alteration in CKD-MBD (48).

Most of the major studies that assessed the effect of FGF23 on the myocardium and vessels did not assess circulating levels of αKlotho. However, it is believed that the deleterious effects on tissues of FGF23 may be attenuated by αKlotho replacement. In the interaction between circulating FGF23 and soluble αKlotho, the latter seems to modulate the effect of the former in three possible ways. First, both may have independent but opposite effects on the myocardium, with FGF23 being harmful and αKlotho protective. This hypothesis, although supported by several studies, does not seem reasonable as it is known that αKlotho and FGF23 are part of a common physiological pathway (18).

Second, soluble αKlotho binds to circulating FGF23, and by inactivation or increasing its renal clearance, blocks the effect of FGF23 on the myocardium. In this case, soluble αKlotho would act as a decoy receptor and bind to excess circulating FGF23 to block its action (18).

Third, soluble αKlotho may serve as a circulating bona fide cofactor for FGF receptors in tissues that do not express membrane αKlotho, like the myocardium. Thus, soluble αKlotho may form complexes with myocardial FGFR1c, favoring FGF23 binding to it in detriment of FGFR4 binding. This change in which FGF receptor FGF23 binds may change the intracellular signaling and resulting effects (18). Recently, Chen et al. confirmed the ability of soluble αKlotho to bind FGF23 and FGFR1c and proposed that the pleiotropic anti-aging effects of soluble αKlotho were dependent on FGF23 (69).

## Conclusion

The CKD-MBD, characterized by the triad of hyperphosphatemia, high FGF23 levels and αKlotho deficiency is central to the pathophysiology of CKD-related cardiomyopathy, so that it can be considered part of the CKD-MBD puzzle. Consequently, the understanding of the individual participation of each component of this triad, as well as the interaction between them, is fundamental for the full clarification of uremic cardiomyopathy pathophysiology and represents the next therapeutic frontier.

Despite the widespread use of phosphorus binders, achieving target phosphate levels is still a major clinical challenge in patients on dialysis. Moreover, phosphate control treatment in this patient group has not been effective in reducing mortality. This may be explained by the fact that serum phosphate levels do not reflect the overall phosphate balance, and by the fact that conventional phosphate control treatment in patients on dialysis is a very late treatment attempt for a disorder that may have set in decades earlier in their pre-dialysis period.

In conclusion, there is a need for a paradigm shift in the treatment of hyperphosphatemia, and new therapeutic options that antagonize the deleterious effects of FGF23 and enhance the protective effects of soluble αKlotho may emerge in the future.

## Author contributions

PGAS, HS-P, and RdP contributed to development of the concepts and design of the review article. PGAS wrote the manuscript and prepared the figures. HS-P and RdP reviewed the manuscript.

### Conflict of interest statement

The authors declare that the research was conducted in the absence of any commercial or financial relationships that could be construed as a potential conflict of interest.
